# Amplification of Trial-to-Trial Response Variability by Neurons in Visual Cortex

**DOI:** 10.1371/journal.pbio.0020264

**Published:** 2004-08-24

**Authors:** Matteo Carandini

**Affiliations:** **1**Smith-Kettlewell Eye Research Institute, San FranciscoCaliforniaUnited States of America

## Abstract

The visual cortex responds to repeated presentations of the same stimulus with high variability. Because the firing mechanism is remarkably noiseless, the source of this variability is thought to lie in the membrane potential fluctuations that result from summated synaptic input. Here this hypothesis is tested through measurements of membrane potential during visual stimulation. Surprisingly, trial-to-trial variability of membrane potential is found to be low. The ratio of variance to mean is much lower for membrane potential than for firing rate. The high variability of firing rate is explained by the threshold present in the function that converts inputs into firing rates. Given an input with small, constant noise, this function produces a firing rate with a large variance that grows with the mean. This model is validated on responses recorded both intracellularly and extracellularly. In neurons of visual cortex, thus, a simple deterministic mechanism amplifies the low variability of summated synaptic inputs into the large variability of firing rate. The computational advantages provided by this amplification are not known.

## Abstract 2

A simple model accounts for the high variability of firing rates observed in responses of cortical neurons to visual stimuli

## Introduction

In the primary visual cortex (V1), different trials of presentation of an identical stimulus yield highly variable firing rates (Heggelund and Albus 1978). This trial-to-trial variability is not inherited from subcortical inputs, as these respond in a much more consistent fashion (Kara et al. 2000). Instead, variability has been related to spontaneous variations in cortical state (Arieli et al. 1996; Buracas et al. 1998; Tsodyks et al. 1999; Kenet et al. 2003). These variations may reflect the perceptual effects associated with a stimulus, rather than the presence of the stimulus itself (Ress and Heeger 2003).

**Figure 1:**
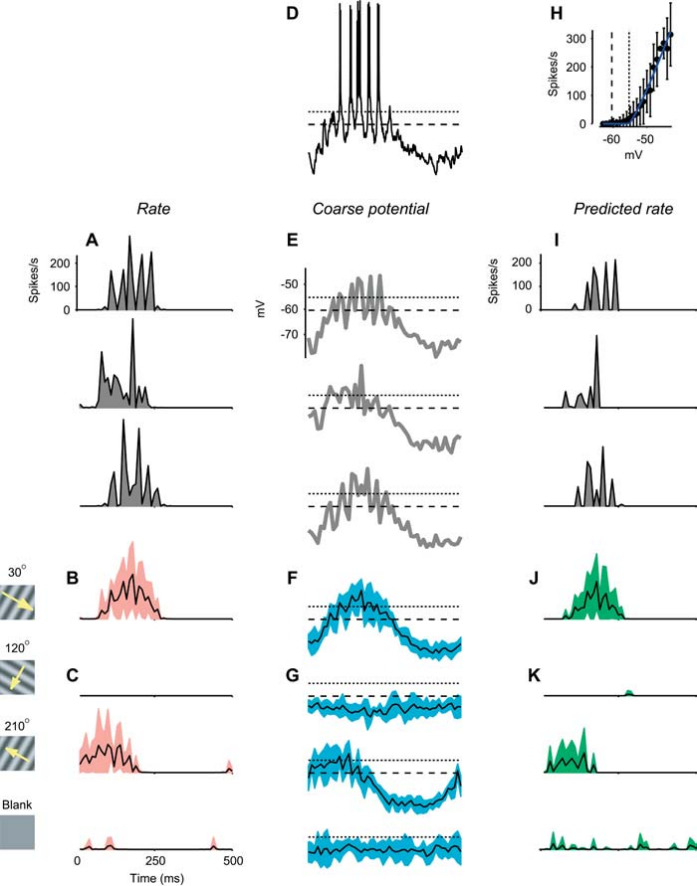
Variability in the Responses of a Simple Cell (A) Firing rate in response to a cycle of an optimal drifting grating. Three trials are shown. (B) Firing rate averaged over seven trials. Shaded area indicates 2 s.d. (C) Same, for three other stimuli: a grating drifting in the orthogonal direction (top), a grating drifting in the opposite direction (middle), and a blank stimulus (bottom). (D) Membrane potential trace measured for the first cycle. Dashed line is resting potential *V_rest_.* Dotted line is firing threshold *V_thresh_* (from [H]). (E–G) As in (A–C), for coarse potential. (H) Relation between firing rate and coarse potential. Curve is fit of rectification equation. (I–K) As in (A–C), for predictions of rectification model.

**Figure 2:**
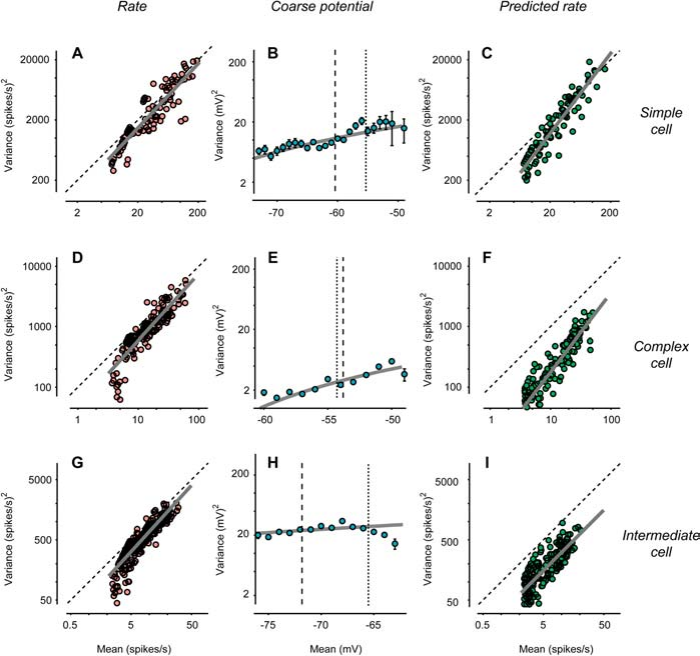
Relation between Response Variance and Mean for Three Cells (A) Variance versus mean for firing rate of the simple cell in Figure 1 measured with 13 stimuli (the four in Figure 1 plus nine additional orientations). Line is linear regression. Diagonal line is prediction for a Poisson process. (B) Variance versus mean for coarse potential. Error bars are 2 s.d. Curve is linear fit to standard deviation versus mean. Dashed line is resting potential *V_rest_.* Dotted line is firing threshold *V_thresh_.* (C) Variance versus mean for firing rate predicted by the rectification model. Details as in (A). (D–F) As in (A–C) for a complex cell. (G–I) As in (A–C) for a third neuron, whose behavior is intermediate between those of simple cells and complex cells.

A key property of trial-to-trial variability is that it depends on the strength of the stimulus: Response variance across trials is approximately proportional to response mean (Tolhurst et al. 1981). An example of this effect can be seen in the responses of a cell in cat V1 to drifting gratings (Figure 1A–1C). Different trials of an identical stimulus elicit firing rates that vary greatly (Figure 1A). As a result, the standard deviation of the firing rates is roughly comparable to their mean amplitude (Figure 1B and 1C). The ratio of variance to mean is close to the value predicted for a Poisson process (Figure 2A, dashed line). For a Poisson process, the variance of the spike counts is equal to the mean. Once spike counts are converted to firing rate by binning in 10-ms windows (i.e., at 100 Hz), the ratio of variance to mean becomes 100. The Poisson-like behavior of firing rates is well known, although reports differ on the exact value of the ratio of variance to mean (Tolhurst et al. 1981; Bradley et al. 1987; Vogels et al. 1989; Geisler and Albrecht 1997; Gur et al. 1997; Reich et al. 1997; Buracas et al. 1998; Kara et al. 2000).

Because the production of firing rates within a neuron introduces remarkably little noise (Calvin and Stevens 1968; Mainen and Sejnowski 1995; Carandini et al. 1996), trial-to-trial variability is thought to arise from the membrane potential fluctuations that result from summated synaptic input (Calvin and Stevens 1968; Stevens and Zador 1998). I have tested this hypothesis by considering membrane potential responses recorded intracellularly in vivo.

## Results

From traces of membrane potential obtained at high temporal resolution (Figure 1D), I obtained an estimate of overall synaptic drive by removing the action potentials and low-pass filtering the resulting traces (Carandini and Ferster 2000; Volgushev et al. 2000). The outcome of this procedure (Figure 1E) is a coarse potential (or “generator potential”; Lankheet et al. 1989) that approximates the synaptic current (Anderson et al. 2000a). This technique allows one to estimate synaptic currents while concurrently recording firing rates.

### Variability of Coarse Potential during Visual Stimulation

We can now consider the mean and variance across trials for coarse potential. The mean, *V_mean_,* is the “signal” reflecting the stimulus-driven synaptic input to the neuron (Figure 1F and 1G, traces). The variance, instead, is the “noise” reflecting the synaptic input's trial-to-trial variability (Figure 1F and 1G, shaded areas).

The variability of potential depended only slightly on stimulus strength. Variance was slightly higher when the stimuli depolarized the cell than when they hyperpolarized it (Figure 1F and 1G). For the example simple cell in Figure 1, standard deviation of potential was 2.8 ± 1.2 mV (s.d.) for *V_mean_* between –70 and –65 mV, and 4.0 ± 1.7 mV for *V_mean_* between –55 and –50 mV. The relation between standard deviation of potential and *V_mean_* can be described by a regression line (*r* = 0.27 ± 0.04, s.d., bootstrap) whose slope is 0.08 ± 0.01 and whose intercept at *V_rest_* = −60.4 mV is 3.3 ± 0.1 mV. Similar values were obtained in the rest of the population (e.g., Figure 2E and 2H): correlation coefficient was *r* = 0.40 ± 0.19 (s.d., *N* = 22), intercept at *V_rest_* was 3.3 ± 1.4 mV, and mean slope was a shallow 0.14 ± 0.09. In occasional cells (such as that of Figure 2H), the standard deviation of potential did not grow monotonically with *V_mean_.*

The ratios of variance to mean seen in membrane potentials were negligible when compared to those seen in firing rate. For the example simple cell, over the entire range of mean potentials the variance of potential grew by less than a factor of four (Figure 2B). By contrast, over the entire range of firing rates the variance of firing rate grew by a factor of almost 100 (Figure 2A). Similar results were obtained in the remaining cells, such as the complex cell of Figure 2D and 2E and the intermediate cell of Figure 2G and 2H. In the last cell, the difference between potential and firing rate was particularly striking, as the former shows a downward slope that is clearly absent in the latter. These differences in variability are meaningful because potential and firing rate were recorded from the same responses to the same set of stimuli. They are not simply due to differences in time scale (Buracas et al. 1998; Kara et al. 2000) because firing rate and potential were sampled at the same resolution (100 Hz).

### Accounting for the Variability of Firing Rate

The origin of the large variability in firing rate lies not in an unforeseen source of noise, but rather in a deterministic mechanism, the nonlinear transformation of potentials into firing rates. This transformation (Figure 1H) can be fitted by a simple rectification model (Granit et al. 1963) describing how firing rate *R* grows with potential *V* once this potential is above a threshold *V_thresh_.* As expected (Anderson et al. 2000b; Carandini and Ferster 2000), this rectification model captures the relation between potential and firing rate (Figure 1H, curve) and can be used to predict the rough features of firing rate both in individual trials (compare Figure 1A and 1I) and in averages across trials (compare curves in Figure 1B and 1C with those in Figure 1J and 1K).

Of course, rectification is not a full account of the transformation between synaptic inputs and firing rates. Indeed, the relationship between firing rate and potential exhibits substantial error bars (Figure 1H). These error bars do not denote noise involved in generating spikes, which is negligible (Calvin and Stevens 1968; Mainen and Sejnowski 1995; Carandini et al. 1996). They simply indicate that (as evident in the Hodgkin–Huxley equations) instantaneous potential is only one of the determinants of firing rate; additional determinants include the membrane potential's recent history (Azouz and Gray 1999) and frequency content (Carandini et al. 1996; Volgushev et al. 2002).

**Figure 3:**
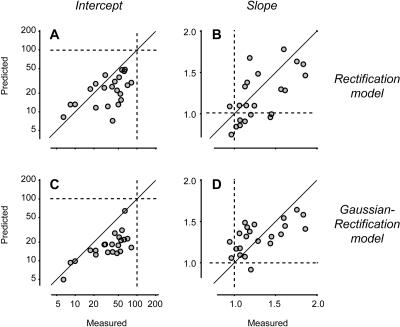
Performance of the Rectification and Gaussian–Rectification Models in Predicting Firing-Rate Variability Distributions of firing-rate variance versus firing-rate mean were fitted with a line in logarithmic scale, corresponding to the equation variance = *a* mean*^b^,* where *a* is the intercept of the line and *b* is the slope of the line. Fitting was performed on the measured distributions (e.g., Figure 2A), on the distributions predicted by the rectification model (e.g., Figure 2C), and on those predicted by the Gaussian–rectification model (e.g., Figure 6B). Dashed lines indicate predictions for a Poisson process. (A) Comparison of measured intercept versus predicted intercept. Diagonal line indicates equality between measured and predicted values. (B) Same, for the slope. (C and D) Same as in (A) and (B), for the predictions of the Gaussian–rectification model.

Despite its simplicity, the rectification model is sufficient to predict the large variability of firing rate, and the increase of firing-rate variance with firing-rate mean. The predicted standard deviation resembles the measured one both in amplitude and in time course (compare shaded areas in Figure 1B and 1C with those in Figure 1J and 1K). Indeed, a plot of variance versus mean for the predicted firing rate (Figure 2C) indicates almost as much variability as that seen for the actual firing rate (Figure 2A). Similar results were obtained in the other example cells (compare Figure 2D to 2F, and 2G to 2I) and in the rest of the population (Figure 3A and 3B). While the rectification model often underestimated the vertical intercept of the line relating mean and variance (Figure 3A), it generally captured the line's slope (Figure 3B). The model, therefore, accounts for the growth of firing-rate variance with the mean.

The reason why the rectification model explains the large variability of firing rate is rather intuitive. Trial-to-trial fluctuations in potential are critical to obtain spikes, because many visual stimuli (such as the 210° grating in Figure 1) elicit a mean potential that barely reaches the firing threshold (Anderson et al. 2000b). Therefore, small fluctuations in membrane potential make the difference between a trial with few or no spikes and one with plenty of spikes. In other words, the firing threshold amplifies small fluctuations in potential into large fluctuations in firing rate.

Perhaps less intuitive is the reason why the rectification model explains the growth of firing-rate variance with firing-rate mean. One may think that a necessary condition for this effect is the growth in potential variance observed with increasing mean potential (Figure 2B). This is not the case: The variance of potential could stay constant or even decrease (as it does for the cell in Figure 2H), and the variance of firing rate would still grow with the mean (Figure 2G).

### Predicting the Variability of Firing Rate

An intuition and a quantitative account for these properties can be obtained by applying the rectification model to an idealized random distribution of potentials, which we take to be Gaussian. Such a Gaussian–rectification model has been used to explain the dependence of mean firing rate on mean synaptic input (Anderson et al. 2000b; Hansel and van Vreeswijk 2002; Miller and Troyer 2002). It resembles a model proposed by Abeles (1982, 1991) to study neuronal integration time.

**Figure 4:**
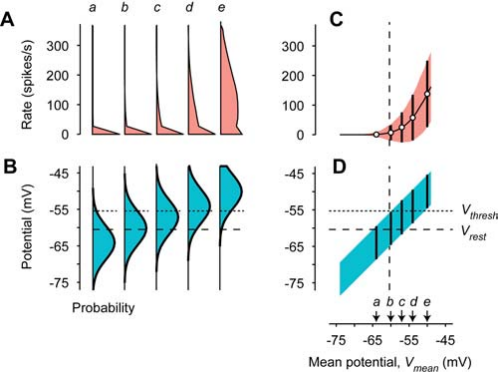
The Gaussian–Rectification Model (A and B) Distributions across trials of model potential *V* (B) and of model firing rate *R* (A) for five values of the mean potential *V_mean_.* Firing rate is obtained from potential by applying the rectification model (Figure 1H). The value for *R* = 0 is shown at 1/3 of veridical height. (C and D) Mean (data points) and standard deviation (error bars) for the distributions in (A) and (B) as a function of mean potential *V_mean_.* Curve and shaded area indicate model predictions for the full range of mean potentials. Arrows indicate the five mean potentials (± 2 mV) used in (A) and (B). Throughout, dashed lines indicate resting potential *V_rest_* and dotted lines indicate firing threshold *V_thresh_.*

In the Gaussian–rectification model, the stimulus determines the mean of the Gaussian (Figure 4B), and the portion of Gaussian that crosses threshold determines the distribution of firing rates (Figure 4A). The mean of the Gaussian is the average potential *V_mean_* evoked by the stimulus at that instant (Figure 4B). The rectification function (Figure 1H) operates on this distribution and determines the distribution of firing rates (Figure 4A): Each potential contributes a firing rate given by the rectification function, with a probability given by the value of the Gaussian at that potential. When mean potential *V_mean_* is low, the Gaussian lies mostly below the threshold *V_thresh_,* so the predicted firing rate is mostly zero (Figure 4A, *a*). When *V_mean_* is higher, however, the tail of the Gaussian that lies above threshold becomes substantially larger, and the distribution of firing rates reaches higher rates (Figure 4A, *e*). The large peak at 0 spikes/s corresponds to the area of the Gaussian that lies below *V_thresh_.*

Such a simple model is sufficient to predict that the variance of firing rate should increase with mean firing rate. As mean potential *V_mean_* increases, the distribution of firing rate becomes broader (Figure 4A), increasing not only in mean but also in standard deviation (Figure 4C). This phenomenon occurs even though in the model the standard deviation of potential is the same at all mean potentials (Figure 4D).

**Figure 5:**
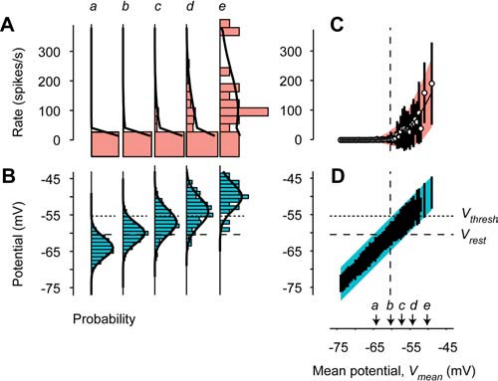
Application of the Gaussian–Rectification Model to the Data from the Example Simple Cell (A and B) Distributions across trials of potential *V* (B) and of firing rate *R* (A) for five values of the mean potential *V_mean_.* Curves are best-fitting Gaussians (B) and predicted distributions of firing rate (A). Bin for *R* = 0 is shown at 1/3 of veridical height (and is three times wider than the others so that area is veridical). (C and D) Mean (data points) and standard deviation (error bars) for the distributions in (A) and (B), as a function of mean potential *V_mean_.* Curve and shaded area indicate model predictions for the full range of mean potentials. Arrows indicate the five mean potentials (± 2 mV) used in (A) and (B). Even a reduced model with constant standard deviation of potential (D, shaded area) predicts a growing standard deviation (A, shaded area).

**Figure 6:**
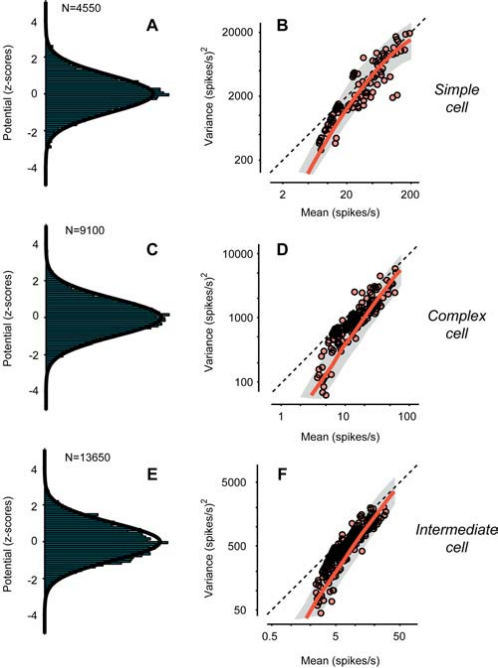
Variability of Potential and Predictions of the Gaussian–Rectification Model for Three Cells (A) Distribution of normalized deviations from the mean (*z*-scores) for the potential of the simple cell in Figure 1 and Figure 2A–2C. These were computed by subtracting from each potential the corresponding mean potential *V_mean_* (the abscissa in Figure 2B) and dividing by the standard deviation (the square root of the ordinate in Figure 2B). The results were cumulated. The curve is a normal Gaussian. (B) Variance versus mean for firing rate for the same cell and its prediction by the Gaussian–rectification model. Data points are same as Figure 2A. Red curve: prediction of Gaussian–rectification model Shaded area: region where the Gaussian–rectification model predicts the occurrence of 75% of the points. Line is linear regression. (C and D) Same as (A) and (B) for the complex cell in Figure 2D–2F. (E and F) Same, for the intermediate cell in Figure 2G–2I.

The main assumption of the model, that of a Gaussian distribution of potentials, is generally borne out by the data. In most cells, the distribution of potential is close to a Gaussian, especially at the lowest values of mean potential, where spiking seldom occurs (Figure 5B). For the example simple cell, the distribution of *z*-scores (the difference between potential and mean potential, normalized by the standard deviation at that potential) appears remarkably Gaussian (Figure 6A). Similar results were obtained in the other cells (e.g., Figure 6C and 6E), although in some cells the tails of the distributions exceeded those of a Gaussian, and a large skewness clearly favored the more depolarized tails (not shown). A Gaussian distribution of potentials is commonly predicted in the theoretical literature (e.g., Svirskis and Rinzel 2000; Amemori and Ishii 2001; Rudolph and Destexhe 2003). It would be expected in a passive membrane summating many independent, high-rate presynaptic spike trains (Rice 1944; Tuckwell 1988).

The Gaussian–rectification model has four parameters. Three of these parameters describe the rectification stage and are thus fully constrained by the measured relationship between potential and firing rate (Figure 1H). The remaining parameter, the standard deviation of the Gaussian, σ, was obtained from maximum likelihood estimation, i.e., by searching for the standard deviation that maximized the probability of observing the distributions of firing rate (Figure 5A). The result, σ = 4.6 mV, slightly overestimates the standard deviation observed for low mean potentials, but correctly estimates it at higher mean potentials (Figure 5D, compare shaded area to error bars).

The model predicts the main features of the distributions of firing rate (Figure 5A). It predicts that when mean potential is low (e.g., *V_mean_* = −64 mV; Figure 5A, *a*), the firing rate is always zero, whereas larger mean potentials yield a distribution of firing rates that spans values from 0 to 300 spikes/s (e.g., *V_mean_* = −54 mV; Figure 5A, *d*). Deviations from the predictions are largest where they are least significant, i.e., at high firing rates for the high values of *V_mean_* (e.g., *V_mean_* = −50 mV; Figure 5A, *e*). These high values were achieved seldom; for example, only 21 data points were obtained at *V_mean_* = −50 mV (Figure 5A, *e*), compared to 273 at *V_mean_* = −54 mV (Figure 5A, *d*) and 1,575 at *V_mean_* = −64 mV (Figure 5A, *a*).

In fact, the model closely predicts both the firing rate's mean and standard deviation (Figure 5C). It predicts the two key effects of increasing mean potential: (1) an increase in the firing rate's mean (as a power law; Anderson et al. 2000b; Hansel and van Vreeswijk 2002; Miller and Troyer 2002), and (2) an increase in the firing rate's standard deviation.

Crucially, the model closely predicts how firing-rate variance depends on firing-rate mean (Figure 6B, red curve). Because of noise in the estimation of variance from a limited number of measurements (in this experiment, seven trials), the data are not expected to fall exactly on the model's prediction; Monte Carlo simulations with a matched number of trials determined the area in which 75% of the observations are predicted to fall (Figure 6B, gray area). Similar results were obtained in the remaining cells of the population, except that the model has a mild tendency to underestimate the intercept and overestimate the slope of the relation between variance and mean (Figure 3C and 3D).

Overall, the Gaussian–rectification model applied to the trace of mean potential performed as well as the rectification model applied to the individual traces of potential. Both models underestimated the intercept of the lines fitted to the relationship between firing-rate variance and mean: the rectification model by 25 ± 42% (Figure 3A), and the Gaussian–rectification model by 44 ± 26% (Figure 3B). Both models correctly estimated the slope of the line (the growth in variance with increasing mean), with insignificant errors of 0.10 ± 0.25 for the rectification model (Figure 3C), and −0.01 ± 0.22 for the Gaussian–rectification model (Figure 3D). This performance is remarkable, given that the Gaussian–rectification model replaces detailed knowledge of potential in individual trials with just one free parameter, the standard deviation σ of potential.

These results illustrate how the key element in producing the steep growth in firing-rate variance observed with growing stimulus strength is the nonlinear transformation between potential and firing rate (Figure 1H). Indeed, the model was intentionally implemented with the constraint that the standard deviation of potential, σ, be constant. This constraint serves to demonstrate that a mild growth in variance of potential (Figure 5D, error bars) is not necessary to produce the steep growth in firing-rate variance (Figure 5C, error bars).

### Variability of Responses to Current Injection

The predictions of the Gaussian–rectification model apply to any neuron that meets minimal criteria: a relationship between synaptic input and firing rate that is monotonic and includes a threshold, and noise in the input that has a Gaussian distribution.

As an example, let us consider a neuron that is closer to biological reality than the Gaussian–rectification model, one that receives currents (not potentials) in its input and produces individual spikes (not continuous firing rates). In particular, consider an enhanced integrate-and-fire neuron, where each spike is accompanied by a temporary increase in spike threshold and by the entry of calcium, which in turn determines an after-hyperpolarization potassium current (see Materials and Methods).

**Figure 7:**
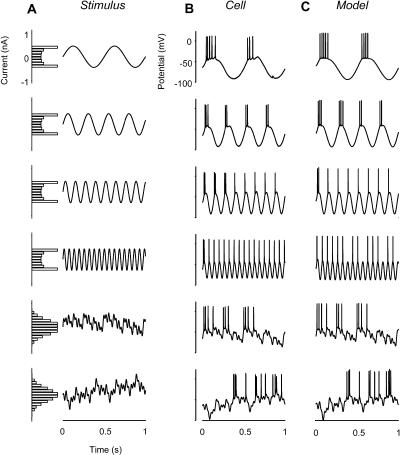
Responses of a Regular-Spiking Neuron in the Visual Cortex to Current Injection, and Predictions by an Enhanced Integrate-and-Fire Model Neuron (A) Injected currents were sinusoids or noise waveforms. Noise was obtained by summing eight sinusoids with incommensurate frequencies. (B) Membrane potential responses of a regular-spiking neuron (cell 19s2, experiment 4) recorded with sharp electrodes in a study of guinea pig visual cortex in vitro (Carandini et al. 1996). (C) Predictions of an enhanced integrate-and-fire neuron model fine-tuned to resemble the responses of the cell.

To ensure realism, I fitted the model parameters to responses to injected currents of a regular spiking neuron. This neuron was recorded in vitro in the visual cortex of the guinea pig, in the near absence of synaptic inputs (Carandini et al. 1996). The injected currents include sinusoids (Figure 7A, top four panels) and approximately Gaussian-distributed noise (Figure 7A, bottom panels). Once its parameters are appropriately tailored, the enhanced integrate-and-fire model accurately predicts the cell's responses, both in the subthreshold membrane potential waveforms and in the timing of individual spikes (Figure 7B and 7C).

**Figure 8:**
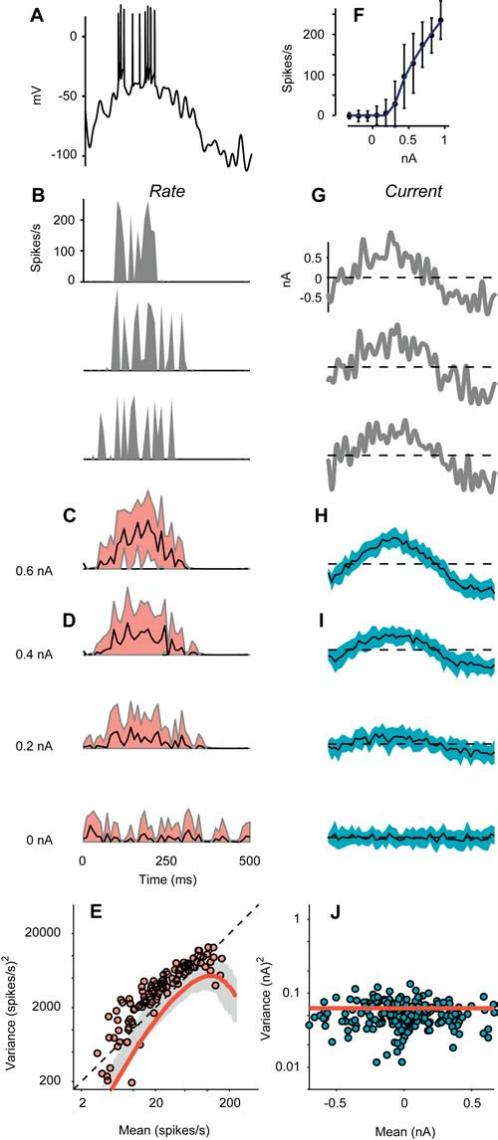
Variability in the Responses of the Spiking Model Neuron (A) Response of the model neuron to a 0.6-nA sinusoidal current in the presence of Gaussian noise (s.d. 0.25 nA). (B) Corresponding firing rate. Three trials are shown. (C) Firing rate averaged over 16 trials. Shaded area indicates 2 s.d. (D) Same, for three other stimuli: a 0.4-nA sinusoid (top), a 0.2-nA sinusoid (middle), and noise alone (bottom). (E) Variance versus mean for firing rate. Diagonal line is prediction for a Poisson process. Red curve: prediction of Gaussian–rectification model, with no parameters allowed to vary to fit the data. Shaded area: region where the Gaussian–rectification model predicts the occurrence of 75% of the points. (F) Relation between firing rate and injected current. Curve is fit of rectification equation. (G–I) As in (B–D), for injected current. (J) Variance versus mean for injected current.

Just as predicted, this spiking neuron responds to noisy injected currents with a firing rate whose variance grows with the mean (Figure 8). To simulate the synaptic drive to a simple cell recorded in vivo (Figure 1A–1D) I injected sinusoidal currents, to which I added Gaussian noise. The model responses (Figure 8A–8D) resemble those seen in vivo (Figure 1A–1D). The firing rate is highly variable (Figure 8B), with a standard deviation that is roughly comparable to the mean (Figure 8C and 8D), even though the standard deviation of the injected current is constant (Figure 8H and 8I). In fact, for firing rate the variance grows proportionally to the mean (Figure 8E), even though for injected current the variance is constant (Figure 8J).

The Gaussian–rectification model captures the essence of this behavior. Once it is given the standard deviation of the noise and the relationship between injected current and firing rate (Figure 8F), the Gaussian–rectification model makes a parameter-free prediction of the relationship between variance and mean (Figure 8E, curve). This prediction is not perfect (it consistently underestimates firing-rate variance), but it does capture the most important behavior: that variance grows with the mean for firing rate (Figure 8E, curve) but not for injected current (Figure 8J, horizontal line).

Similar results were obtained when the stimulus parameters were changed to simulate synaptic inputs to a complex cell, or when the parameters of the spiking neuron were changed to simulate other cells measured in vitro, or even chosen randomly within reasonable bounds. As predicted, as long as the relationship between synaptic input and firing rate involved a threshold and the input noise was Gaussian, the variance grew with the mean for firing rate but not for injected current.

### Role of Firing-Rate Encoder

**Figure 9:**
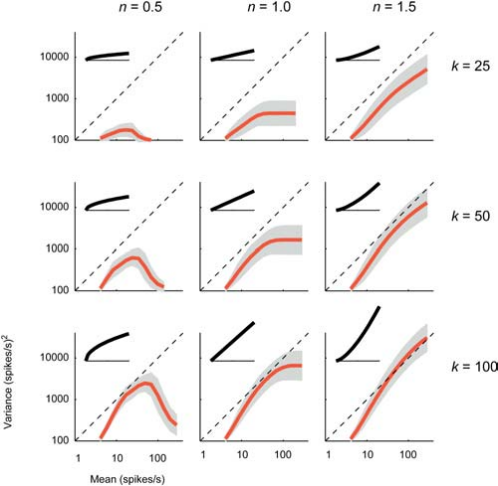
Role of Parameters of Gaussian–Rectification Model The standard deviation of potential was set to σ = 1, so that the shape of the curves relating firing-rate variance to firing-rate mean depends entirely on the gain *k* and the exponent *n* of the curves relating firing rate to membrane potential. The effects of these two parameters are explored: varying *n* (columns) and varying *k* (rows). Red curves: predictions of the Gaussian–rectification model; shaded areas: regions where the model predicts the occurrence of 75% of the points. Insets illustrate the corresponding curves relating firing rate to membrane potential.

Having validated the Gaussian–rectification model, we can now investigate the role of its parameters in determining the curves relating firing-rate variance and mean (Figure 9). The model has four parameters (see Materials and Methods): (1) the standard deviation σ of potential, (2) the firing threshold, *V_thresh_,* (3) the gain *k* of the relationship between firing rate and potential above threshold, and (4) the exponent *n* of this relationship. For the purpose of studying the model, we can assume, without loss of generality, that potential is unitless and has standard deviation σ = 1. Then, because *V_thresh_* can only determine the range of firing rates that is achieved, only *k* and *n* control the shape of the variance versus mean curves (Figure 9).

The gain *k* controls curve position, and the exponent *n* controls curve shape (Figure 9). Increasing the gain *k* lifts the curves upward by twice as much as it shifts them rightward (Figure 9, rows). These shifts occur because variance grows with *k* ^2^ and mean grows with *k.* Decreasing the exponent *n* causes the curves to saturate (Figure 9, columns): The variance saturates to a plateau if *n* = 1 (Figure 9, middle), and it reaches a maximal value and then decreases if *n* < 1 (as in Figure 9, left). Saturation occurs because when potential goes well above threshold, increases in mean potential cease to reveal ever larger portions of the Gaussian. If the curves relating firing rate to potential saturate (*n* < 1), variations in potential are compressed into proportionally ever smaller variations in firing rate; the opposite occurs if the curves expand (*n* > 1).

This analysis predicts that it should be fairly common for the firing-rate variance to saturate at high firing rates, possibly showing a plateau or even a decrease. Indeed, in the sample of V1 neurons recorded intracellularly, exponents are typically close to unity (*n* = 1.1 ± 0.6). Accordingly, a mild form of saturation is common in the plots of firing-rate variance and mean (Figure 6B). To quantify the saturation, however, one needs reliable estimates of firing-rate variance. These estimates are not very reliable in the intracellular sample, which typically involves only a few hundred spikes per cell, leading to large clouds of points in the scatters of variance versus mean (Figure 6B).

### Variability of Extracellularly Recorded Firing Rates

To test the model's prediction rigorously, I considered a set of V1 responses obtained with extracellular recordings. Thanks to the large number of spikes (commonly >4,000 per cell), measurements in this dataset yield more precise estimates of firing-rate variance over a wider range of firing rates than are available in the intracellular sample.

**Figure 10:**
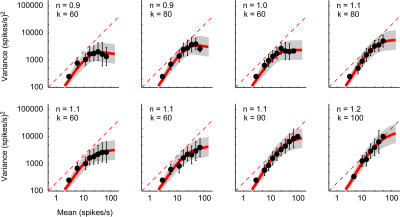
Relationship between Variance and Mean for Eight Cells Recorded Extracellularly in Cat V1, and Fits by the Gaussian–Rectification Model For each mean firing rate, data point and error bars indicate mean ± 1 s.d. of the observed variance. Red curves and shaded areas are predictions of the model. Values of exponent *n* and gain *k* are reported next to each graph. Cells are arranged in order of increasing exponent *n.*

An analysis of firing-rate variance versus mean for these extracellularly recorded cells supports the predictions of the model (Figure 10). Extracellular data do not afford independent estimates of gain *k* and exponent *n* of the transformation of potential into firing rate. I thus first computed the model predictions for a variety of combinations of *k* and *n* (such as those shown in Figure 9). I then made Bayesian estimations of the values of *k* and *n* that maximize the likelihood of the data, while imposing a broad prior for *n* = 1.1, the median value measured intracellularly. The quality of these two-parameter fits was excellent (Figure 10), of higher quality than could be obtained by fitting a line, the two-parameter “model” commonly used to describe data of this kind (Figure 2A). Moreover, a number of cells exhibited the saturation in variance predicted by the model. The eight representative cells shown in Figure 10 are arranged in order of increasing exponent *n.* The first three (*n* = 0.9 to 1.0) show evident saturation in firing-rate variance as mean firing rate increases. The remaining five show a milder saturation, as expected from their higher exponents (*n* = 1.1 to 1.2). Saturation was common, as the median *n* was 1.06, with *n* < 1 in 13/37 cells. Yet to my knowledge, except for an anecdotal account (Mechler 1997), this common property had not been previously reported. It constitutes further support for the usefulness of the Gaussian–rectification model.

## Discussion

We have seen that a large amplification takes place between the trial-to-trial variability of synaptic input and that of firing rate: The variance of synaptic input is small compared to the dynamic range, and it is roughly constant. The amplification of variability arises from the threshold in the transformation of synaptic input into firing rate.

A Gaussian–rectification model attributes this amplification to very simple causes: approximately constant Gaussian noise in the input, and rectification due to threshold in the output. It indicates that firing-rate variance would grow with the mean even if the variance of synaptic input were constant. Both of the assumptions of the model, constant Gaussian noise and rectification, are borne out by the data. These assumptions are rather minimal, so they are naturally satisfied by more realistic models. For example, a realistic integrate-and-fire model behaves as predicted: Once it is given constant Gaussian noise in the input, it produces a firing-rate variance that grows with the firing-rate mean. Further support for the Gaussian–rectification model comes from its novel, and correct, prediction that firing-rate variance should saturate at high firing rates. In confirming this prediction I showed that the model can be used to account for variability in firing rate without knowledge of cellular properties. The extension to extracellular data is important because extracellular methods constitute the norm in visual neurophysiology, especially in awake animals, and are the ones used in previous studies of firing-rate variability.

These results further lengthen a list of properties of V1 neurons that are simply explained by the firing threshold. In addition to the amplification of trial-to-trial variability demonstrated here, these include the sharpening of tuning for stimulus direction and orientation (Jagadeesh et al. 1993; Carandini and Ferster 2000; Volgushev et al. 2000), the power-law behavior of firing rate at low contrast (Heeger 1992; Anderson et al. 2000b; Hansel and van Vreeswijk 2002; Miller and Troyer 2002), and even the establishment of the dichotomy between simple and complex cells (Carandini and Ferster 2000; Mechler and Ringach 2002; Priebe et al. 2004). It is remarkable that a mechanism as simple as the firing threshold can determine phenomena that might prima facie require more complex explanations at the level of the network.

### Limitations of the Approach

One limitation of this study lies in the use of coarse potential. Coarse potential is not completely independent of firing rate: Even when spikes are removed and the traces smoothed, there is still a likely contribution of active conductances that has not been removed. Fortunately, this limitation strengthens my observation that coarse potential is not nearly as variable as firing rate: Any unwanted remaining echo of the spikes would make coarse potential more similar to firing rate and, thus, more variable. Therefore, in reality the variance of the actual synaptic input might be even less dependent on the mean than appears, for example, in Figure 2B, 2E, and 2H. A partial control for these effects would be to perform some of the measurements while blocking spikes. However, blocking spikes would prevent the key measurements of this study, which require concurrent measurement of firing rate and estimation of synaptic input.

Another limitation of the approach is that I have mostly considered firing rates, not individual spikes. Unlike firing rates, individual spikes can occur only in integer numbers and are separated by refractory periods. These properties can become relevant to response variability, for example, if firing rates become so high that refractory period becomes a limiting factor (Kara et al. 2000). Such concerns are assuaged by the realistic integrate-and-fire model (Figure 7), which shows an increase of firing-rate variance with the mean similar to that predicted by the Gaussian–rectification model. As to the saturation in firing-rate variance that was observed in some neurons, it invariably occurred at firing rates much lower than predicted from the refractory period.

A more serious limitation of coarse potentials and firing rates is that they make sense only in a limited range of time windows. The windows should be long enough to be able to contain more than one spike, and short enough that mean potential is approximately constant within the window. An informal analysis of the effect of time window indicates that a range of 5–20 ms is satisfactory. This range, however, might be appropriate only for V1 neurons; further investigations are required before applying these methods elsewhere.

Finally, a broader limitation of this work is that it concentrates on variability across trials, with little bearing on another form of variability, the one observed within trials in the irregularity of spike trains (Softky and Koch 1993; de Ruyter van Steveninck et al. 1997; Reich et al. 1997; Troyer and Miller 1997; Buracas et al. 1998; Shadlen and Newsome 1998; Stevens and Zador 1998). Thanks to recent advances, however, the cellular origins of this form of variability have been largely explained (Reich et al. 1997; Stevens and Zador 1998). In particular, it is now clear that high variability within trials is to be expected if neurons receive synaptic inputs with slow temporal correlation (Svirskis and Rinzel 2000). In fact, variability within trials is most evident with visual stimuli that provide a roughly stationary response, being greatly diminished with richer stimuli, which elicit highly precise responses (Bair and Koch 1996; Reich et al. 1997; Buracas et al. 1998). Conversely, trial-to-trial variability is endemic, being present regardless of type of visual stimulus (Reich et al. 1997; Buracas et al. 1998).

### Implications for Cortical Processing

What computational advantage might cortical neurons derive by amplifying the variability that they receive in their input? Why reduce the signal/noise ratio? To answer these questions, it might help to clarify the sources of “signal” and “noise.” The main source of variability in synaptic inputs to a V1 neuron is likely to be intracortical because thalamic responses are half as variable (Kara et al. 2000). Variability thus results largely from ongoing cortical activity (Arieli et al. 1996; Buracas et al. 1998; Tsodyks et al. 1999; Kenet et al. 2003). It appears to us as noise simply because it is not synchronized with stimulus onset. By contrast, the mean across trials of potential or firing rate constitutes a signal that is driven entirely by the stimulus.

The results of this study suggest that threshold affects the interaction between stimulus-driven activity and ongoing activity, turning it from additive to multiplicative. At the level of firing rates, this interaction is largely multiplicative because the variance of firing rate grows proportionally to the stimulus-driven mean firing rate. At the level of synaptic inputs, instead, this interaction is nearly additive because the variance of potential barely depends on the stimulus-driven mean potential. Indeed, additivity has been seen between local field potentials and ongoing voltage-sensitive dye signals (Arieli et al. 1996). We have seen that the rectification due to firing threshold is single-handedly responsible for the variability of firing rate and is, thus, responsible for turning a largely additive interaction into a multiplicative interaction.

It is thus conceivable that the computational role of firing threshold is to multiply stimulus-driven responses by ongoing cortical activity, i.e., to multiply what we call “signal” by what we call “noise.” What may appear as lowering the signal/noise ratio can in fact be seen as a useful process, one that progressively amplifies the ongoing activity that ultimately guides our actions.

## Materials and Methods

### 

#### Data acquisition in vivo

Measurements in vivo were obtained in paralyzed, anesthetized cats. Methods for animal preparation and maintenance have appeared elsewhere (Carandini and Ferster 2000) and were approved by the Animal Care and Use Committees at Northwestern University and at the Smith-Kettlewell Eye Research Institute.

The 22 cells recorded intracellularly belong to a sample that has been analyzed in two previous studies by Carandini and Ferster (2000) and by Anderson et al. (2000a). These studies describe in detail the recording methods, which involved the whole-cell patch technique. The electrical noise associated with this technique is commonly <0.1 mV (as judged from records obtained after losing the patch). From the sample I excluded a few cells that produced less than ten spikes per block of stimuli, or that failed to satisfy other minimal requirements (firing rate >2 spikes/s, spike height >10 mV). Stimuli were optimal gratings drifting in 12 directions in 30° intervals, and a blank screen of uniform gray. The resting potential *V_rest_* was taken as the mean potential measured with the blank screen. Coarse potential traces were obtained from traces of membrane potential sampled at 4 kHz by removing spikes (Lankheet et al. 1989) and by applying a low-pass filter with a cutoff of 50 Hz (Carandini and Ferster 2000; Volgushev et al. 2000). The same low-pass filter was applied to spike trains sampled at 4 kHz to yield firing rate. Both coarse potential and firing rate were subsampled at 100 Hz.

The 37 neurons recorded extracellularly are part of a study of the organization of receptive fields and suppressive surrounds in area V1 (Bonin et al. 2003). This dataset was chosen because it involved lengthy experiments that yielded many thousands of spikes per cell at a variety of firing rates. Recordings were made with quartz-coated platinum/tungsten microelectrodes; methods for data acquisition and animal maintenance have been described by Freeman et al. (2002). Stimuli were drifting gratings presented at the optimal orientation, spatial frequency, and temporal frequency, and enclosed in one of 66 possible windows. The windows were stationary square gratings with variable period and orientation. Stimuli typically lasted 2 s, and each block of stimuli was typically repeated three to six times. Firing rates were extracted from the spike train by low-pass filtering at 50 Hz and were subsampled at 100 Hz.

#### Data acquisition in vitro

Measurements in vitro were made with sharp intracellular electrodes from slices of guinea pig visual cortex. Methods for this preparation were approved by the Animal Care and Use Committee at New York University. The cells are part of the dataset presented by Carandini et al. (1996); the cell in Figure 7 is the one whose responses are extensively illustrated in that study (cell 19s2).

#### Rectification model

The relation between potential *V* and firing rate *R* (e.g., Figure 1H) was fitted with an extension of the rectification model (Mechler and Ringach 2002), where *R*(*V*) = *k*[*V*−*V_thresh_]^*n*^_+_* , with [.]_+_ indicating rectification, *k* a proportionality factor, and *n* an exponent. Fitted parameters were *V_thresh_* = −55.3 mV, *k* = 16.7, and *n* = 1.2 for the simple cell in Figure 1, and *V_thresh_* = −46.6 ± 10.5 mV, *k* = 12.4 ± 7.9, and *n* = 1.1 ± 0.6 for the whole intracellular population (*N* = 22). The distance between *V_thresh_* and *V_rest_* was 5.1 mV for the simple cell in Figure 1, and 8.0 ± 4.2 mV for the population.

#### Gaussian–rectification model

The mean potential *V*_mean_ in response to a stimulus was defined as the mean across trials of coarse potential.

In the Gaussian–rectification model, the probability of observing a firing rate *r* (Figure 4A) given a mean potential *V_mean_* is



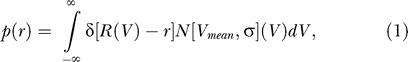



where *R*(*V*) is the relation between firing rate and potential *V* (Figure 1H), and *N*[*V_mean_, σ*] is the probability distribution of potential (Figure 5B), a Gaussian with mean *V_mean_* and standard deviation *σ.* The value of *p*(*r*) depends on whether *r* is zero or positive:



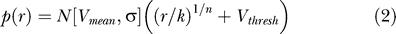



for *r* > 0, and



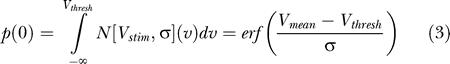



for *r* = 0. The first expression is simply the value of the Gaussian for *V* = *R^−1^*(*r*). The second expression is the area of the portion of Gaussian that is below threshold (*erf* is the error function).

These expressions allow maximum likelihood estimation of model parameters from measured firing rates. When parameters of the relation between firing rate and potential *R*(*V*) are obtained independently (in intracellular recordings; Figure 1H), the only free parameter was the standard deviation σ of potential. Across the intracellular population, the average value of σ obtained by the fits was 5.4 ± 2.0 mV (s.d., *N* = 22). The required σ was always larger (by 2.1 ± 1.5 mV) than the standard deviations observed when *V_mean_* = *V_rest_,* but it was comparable (larger by only 0.9 ± 1.6 mV) to the standard deviations observed when *V_mean_* = *V_thresh_.*

#### Statistics

Let *V*_mean_(*t*) be the mean potential at time *t* from stimulus onset. Because the sample rate is 100 Hz, each time sample corresponds to a 10-ms interval. Of course, *V_mean_*(*t*) depends on the stimulus. To simplify the notation, however, consider the case of a single stimulus.

Distributions for potential at a given mean potential (Figure 5B) were computed as follows: (1) Select a value of interest, *v* (e.g., *v* = −55 mV; Figure 5B, *a*); (2) find the times (*t_k_*) when the mean potential *V_mean_*(*t_k_*) is within 2 mV of *v;* (3) pooling across trials *j,* look at the distribution of potential [*V_j_*(*t_k_*)] (e.g., Figure 5B, *a*).

Distributions for *z*-scores (normalized deviations from the mean) of potential (Figure 6A, 6C, and 6E) were computed as follows: (1) Divide the range of values of *V_mean_* in 1-mV intervals, with centers (*v_i_*); (2) for each interval *i,* find the set of times (*t_ik_*) when the mean potential is in the *i*-th bin; (3) pooling across trials *j,* compute *σ_i_,* the standard deviation of *V_j_*(*t_ik_*); (4) transform each *V_j_*(*t_ik_*) into a *z*-score: z*_ijk_*= [*V_j_*(*t_ik_*) − *v_i_*]/*σ_i_;* (5) look at the distribution of (z*_ijk_*) (e.g., Figure 6A).

#### Enhanced integrate-and-fire model

The enhanced integrate-and-fire model was derived in collaboration with Davide Boino (2000) by simplifying a model by Wang (1998). The model neuron has a single compartment with membrane equation



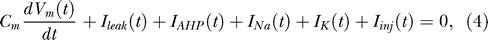



where the currents are:



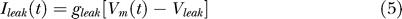





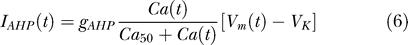





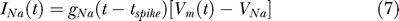





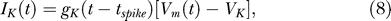



with *t_spike_* the time of the last spike. *Ca*(*t*) is the (unitless) calcium concentration:



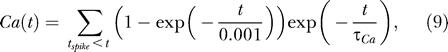



where the sum extends over all spikes with *t_spike_ < t.*

Spikes result from stereotyped conductances *g_Na_*(*t*) and *g_K_*(*t*) derived from Hodgkin–Huxley equations and are scaled to approximate the spikes from the recorded neuron. They occur when *V_m_* exceeds a threshold, which depends on the time since the last spike:



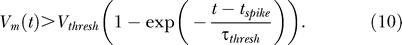



The reversal potentials for sodium and potassium were set to *V_k_* = −80 mV and *V_Na_* = 55 mV. Passive parameters of the membrane (*C_m_* = 120 pF, *g_leak_* = 12.4 nS, *V_leak_* = −60.3 mV) were obtained by fitting the membrane potential responses to sinusoids. The remaining parameters (*g_AHP_* = 23.0 nS, *Ca_50_* = 10, τ*_Ca_* = 200 ms, V_thresh_ = −43.5 mV, τ*_thresh_* = 36 ms) were obtained by a search algorithm aimed at maximizing the quality of the predictions for firing rate.

## Abbreviation

V1: primary visual cortex
